# Alexandrian Cut in Downstream Lignin Valorization to Yield Novel Plasticizers

**DOI:** 10.1021/acscentsci.3c00007

**Published:** 2023-01-31

**Authors:** Maxim V. Galkin, Joseph S. M. Samec

**Affiliations:** † Nanotechnology and Functional Materials, Department of Materials Science and Engineering, Ångstrom Laboratory, Uppsala University, 751 21, Uppsala, Sweden; ‡ Department of Organic Chemistry, Arrhenius Laboratory, Stockholm University, 106 91 Stockholm, Sweden; § Department of Chemistry, Faculty of Science, Chulalongkorn University, Pathumwan, Bangkok 10330, Thailand

Biomass is seen as a renewable alternative to fossil-based carbon sources.^1^ One part of biomass is lignin, a phenolic biomacromolecule. A common approach in lignin valorization is depolymerization that results in the production of complex mixtures of monophenols, that can be used as bulk chemicals for the production of materials.^2^ However, transforming the monophenols has proven difficult. To date, there has been little attention to downstream processing of the generated phenol monomers with few exceptions.^3,4^ Most reports have focused on chemistry directed to the propyl chain of the monophenol, part/full deoxygenations, or Friedel-Craft’s chemistry. The direct activation of Csp^2^–OH is challenging due to the high acidity and reactivity of the phenol hydroxy group and at the same time the rather strong Csp^2^–O bond.^5^ That, in turn, makes this motif challenging to transform catalytically, especially in the presence of the ortho methoxy groups. A common approach to overcome the poor reactivity of the phenol group is to perform a functional group interconversion (FGI).^5^

In this issue of A*CS Central Science*, the groups of Beckham and Stahl used an approach orthogonal to the state-of-the-art by reacting phenol monomers at the phenolic position after an initial functional group interconversion.^6^ This strategy is advantageous with respect to both chemo- and site-selectivity in addition to mitigating the challenges of an undesired phenol in downstream processing. Converting aryl sulfonates using nickel or a combination of nickel- and palladium-catalyzed reductive coupling reactions has been previously reported using stoichiometric reductants such as zinc powder.^7^

**Scheme 1:**
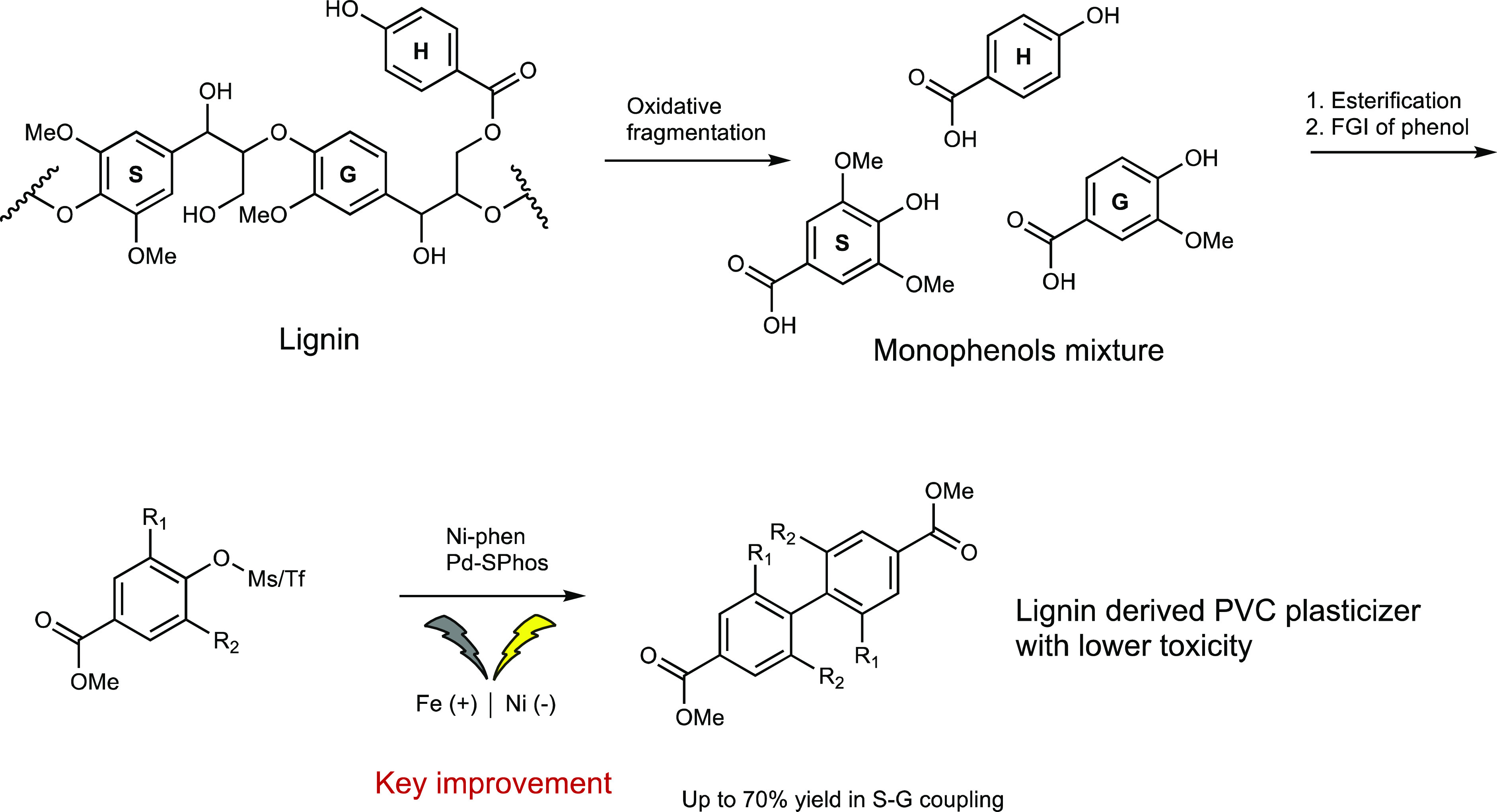
Conversion of Lignin into Pure Biphenyl-4,4′-dicarboxylates via Ni- and Ni/Pd-Catalyzed Reductive Coupling R1 and R2 = H or MeO. G, S, and H are guaiacyl, syringyl, and coumaryl derivatives, respectively.

Lignin-derived monophenols often represent a complex mixture of compounds. First, different biomass sources containing different derivatives of lignin–guaiacyl (G) derivatives are found in all types of biomasses, whereas syringyl (S) derivatives are additionally present in hardwoods, and herbaceous plants such as switchgrasses contain all variants, including coumaryl derivatives (H).^8^ Second, the method of biomass fractionation and lignin deconstruction will increase the number of possible products. For instance, oxidative catalytic fractionation (OCF) of a hardwood will give a mixture of vanillin, and the corresponding vanillic acid and syringyl aldehyde (syringyl acid).^9^ Such mixtures are much more challenging to convert at the phenolic position due to both the steric effects of the neighboring methoxy groups and higher reactivity of the methoxy group in comparison with phenol.^5^ In this work, the authors chose the corresponding ester of the three possible lignin-derived monophenols, that are viable from lignin in a few reaction steps. The authors found that varying the sulfonate group was important for tuning the reactivity. For instance, coumaryl and guaiacyl derivatives were reactive as corresponding mesylates, whereas the syringyl was only reactive when converted to a triflate. Applying high-throughput experimentation, different catalysts were screened, and it was found that a combination of nickel with a phenantroline ligand together with palladium and a biaryl phosphine ligand (SPhos) gave the best results.

The coupling reaction of two aryl sulfonates is formally a reduction. As stated above, the original reactions were performed using zinc powder as a stoichiometric reductant.^7^ From a green chemistry perspective, this is not optimal as the most benign reductant is electrons. The authors were able to substitute the stoichiometric metal as the reductant by applying electrochemistry, where a nickel foam-based cathode provided electrons for the reduction reaction. Using this tandem catalytic system, coupling of two aryl sulfonates to generate biaryls in good yields was achieved. Noteworthy, coupling of two different biaryl compounds with high selectivity was possible where the desired cross coupling product was isolated in 70% yield, even using challenging S-, and G-sulfonates. In many cases the electrochemical system worked better than the stoichiometric system using zinc as the reductant. The authors demonstrate both the possibility of scaling up the reaction and performing it under flow conditions.

The generated biaryl compounds were then applied to the synthesis of a plasticizer for polyvinyl chloride (PVC) and benchmarked to fossil-derived and commercially available di(2-ethylhexyl)-phtalate (DEHP). The developed plasticizer showed comparable to improved properties to DEHP. This approach opens up a new avenue in lignin valorization to achieve high selectivity by reacting on the phenol using a simple FGI and demonstrates upscaling possibilities using flow chemistry and benign electrochemical reduction. Several applications of this methodology are possible in both materials science and fine chemical manufacturing. A future goal will be to find a more atom economical approach to the FGI of the phenol.
